# The dark side of empathy in narcissistic personality disorder

**DOI:** 10.3389/fpsyt.2023.1074558

**Published:** 2023-03-30

**Authors:** Ester di Giacomo, Elena Andreini, Ottavia Lorusso, Massimo Clerici

**Affiliations:** ^1^Department of Mental Health and Addiction, Health Care Trust–IRCCS San Gerardo Monza, Monza, Italy; ^2^School of Medicine and Surgery, University of Milano-Bicocca, Monza, Italy

**Keywords:** empathy, crime, narcissistic personality disorder, psychotherapy, rivalry, mentalization, mindreading

## Abstract

Narcissistic personality disorder is characterized by self-absorption, grandiosity, exploitation of others and lack of empathy. People with that disorder may switch from an overt form, mainly with grandiosity, to a covert presentation, with fears, hypersensitivity and dependence from others. Empathy represents a key point in detecting people affected by narcissistic personality disorder because, even if it is described as reduced, it plays a fundamental role in exploitation and manipulation. A systematic search of Literature without any language or time restriction, was performed combining thesaurus and free-search indexing terms related to Narcissistic personality disorder and empathy and produced 531 results. Fifty-two papers that analyzed possible issues in the empathic attitude of people with narcissistic personality disorder were included in this narrative review. Empathy is the capability of understating and feeling others emotions. It is not a unitary construct and can be distinguished in cognitive and affective. It might be channeled into prosocial and antisocial behaviors. A crucial trait identified in narcissistic empathy is affective dissonance that is closely related to rivalry as part of the dark tetrad (narcissism, machiavellianism, psychopathy, and sadism). Subjects affected by narcissistic personality disorder show greater impairment in affective aspects while their cognitive part of empathy appears preserved. Saving at least the cognitive aspects of empathy may contribute to therapeutic improvement of affective aspects.

## Introduction

Narcissistic personality disorder is characterized by self-absorption, grandiosity, exploitation of others and lack of empathy. The tendency to elicit admiration from others is epitomic, but it is manipulative and finalized to take a personal advantage. Empathy plays a crucial but ambivalent role in people affected by narcissistic personality disorder (NPD), who often misunderstand someone else’s empathic behavior and social assistance.

The rise of narcissism over the generations, as shown by increased scores in questionnaires about that disorder in American college students in the last 25 years, seems typical of western cultures and stresses the importance of analyzing such a phenomenon.

This narrative review aimed at analyzing the interplay between NPD and different aspects of empathy with the goal of a better understanding of antisocial/prosocial behaviors in NPD. Furthermore, implications and treatment options will be discussed.

## Methods

A systematic search of Literature in two main databases (PubMed and Embase), without any language or time restriction, was performed until October 2022 combining thesaurus and free-search indexing terms related to Narcissistic personality disorder and empathy. The review was performed according to PRISMA-ScR and produced 531 results (207 in PubMed and 324 in Embase). Studies that did not describe both narcissism and empathy were excluded. Experimental research would be included if it diagnoses narcissistic personality disorder or analyzes empathy through standardized tests.

**FIGURE 1:**
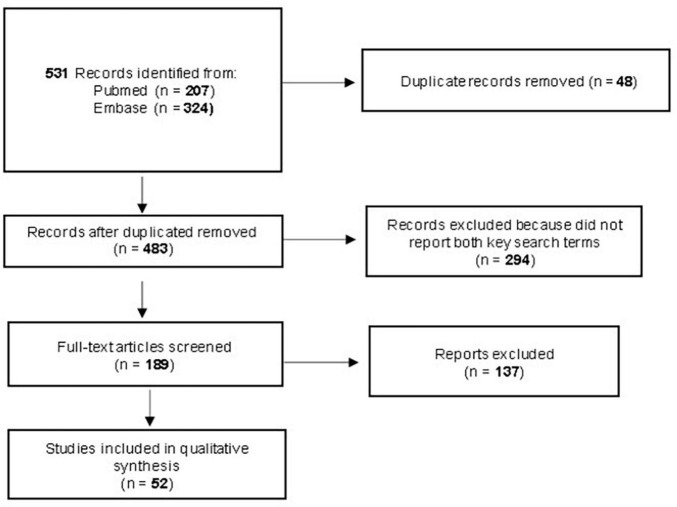
Preferring reporting items for systematic reviews and meta analyses flow diagram.

One-hundred eighty-nine full texts were analyzed and fifty-two articles were included in qualitative analysis (see Figure 1).

## Results

Table 1 provides an overview of the papers included in the qualitative analysis. Most of the manuscripts were published in the last 15 years (46 out of 52).

**TABLE 1:** Studies included in qualitative synthesis.

References	Title	Year	Country	Study type	Empathy measures	Narcissism measures	Other measures	Category
Amiri and Behnezhad (48)	Emotion recognition and moral utilitarianism in the dark triad of personality.	2017	Iran	Cross-sectional study	IAPS	–	SD3	Antisocial behavior
Barry et al. (54)	Self-perceptions of social support and empathy as potential moderators in the relation between adolescent narcissism and aggression.	2014	USA	Cross-sectional study	TEQ	PNI; NPIC	PCS; SSS	Antisocial behavior
Baskin-Sommers et al. (61)	Empathy in narcissistic personality disorder: From clinical and empirical perspectives	2014	USA	Review	–	–	–	Narcissism/empathy correlation
Bilotta et al. (27)	Symptom severity and mindreading in narcissistic personality disorder.	2018	Italy	Cross-sectional study	MAI	SCID-I; SCID-II	SCL90-R; TAS-20	Narcissism/empathy correlation
Blasco-Belled et al. (39)	Vulnerable narcissism is related to the fear of being laughed at and to the joy of laughing at others.	2022	Poland/Spain	Cross-sectional study	–	HSNS	Phophikat-45/9; VIEQ	Narcissism/empathy correlation
Charles (45)	Narcissism, need for power, and social interest	1998	USA	Cross-sectional study	SOI	NPI	SIS; NPS	Antisocial behavior
Christopher et al. (10)	Narcissists- social pain seen only in the brain	2015	USA	Cross-sectional study		NPI	fMRI data analysis; NTS	Neurophysiological aspects
Chukwuorji et al. (4)	Different slopes for different folks: Gender moderates the relationship between empathy and narcissism	2020	Nigeria	Cross-sectional study	IRI	NSS	–	Narcissism/empathy correlation
Decety and Moriguchi (15)	The empathic brain and its dysfunction in psychiatric populations: Implications for intervention across different clinical conditions.	2007	USA	Review	–	–	–	Neurophysiological aspects
Deliè et al. (25)	Self-reported emotional and social intelligence and empathy as distinctive predictors of narcissism.	2011	Slovenia	Cross-sectional study	ESCQ; TSIS; IRI	NPI		Narcissism/empathy correlation
Di Pierro et al. (35)	The role of identity instability in the relationship between narcissism and emotional empathy.	2018	Italy	Cross-sectional study	MET	PNI	RPQ	Narcissism/empathy correlation
Dimaggio et al. (2)	Know yourself and you shall know the other. to a certain extent: Multiple paths of influence of self-reflection on mindreading	2008	Italy/USA	Review	–	–	–	Narcissism/empathy correlation
Drozek and Unruh (29)	Mentalization-based treatment for pathological narcissism.	2020	USA	Review	–	–	–	Therapeutic implication
Fourie (7)	Narcissistic behavior and the successful conservation of ambivalence.	2010	South Africa	Review	–	–	–	Narcissism/empathy correlation
Giacomin and Jordan (22)	Down-regulating narcissistic tendencies: Communal focus reduces state narcissism	2014	Canada	Longitudinal study	–	NPI	RSES; PES	Therapeutic implication
Gojković et al. (43)	Structure of darkness: The dark triad, the “dark” empathy and the “dark” narcissism	2022	Serbia	Cross sectional study	ACME	NARQ	SD3	Antisocial behavior
Hartmann (63)	Psychoanalytic self-psychology and its conceptual development in light of developmental psychology, attachment theory, and neuroscience	2009	USA	Review	–	–	–	Therapeutic implication
Hengartner et al. (28)	Fluid intelligence and empathy in association with personality disorder trait-scores: Exploring the link	2014	Switzerland	Cross-sectional study	RMET; IRI	ADP-IV	DSCT;	Narcissism/empathy correlation
Hepper et al. (26)	Moving narcissus: Can narcissists be empathic?	2014	USA	Cross-sectional study	IRI	NPI	–	Narcissism/empathy correlation
Hepper et al. (56)	Narcissism and empathy in young offenders and non-offenders	2014	United Kingdom	Case-control study	IRI	NPI; SCID-II	–	Antisocial behavior
Heym et al. (46)	Empathy at the heart of darkness: Empathy deficits that bind the dark triad and those that mediate indirect relational aggression.	2019	United Kingdom	Cross-sectional study	QCAE		IAS-A; SD3	Antisocial behavior
Holmes (65)	The technique of partial identification: Waking up to the world	2009	USA	Review	–	–	–	Therapeutic implication
Jankowiak-Siuda and Zajkowski (3)	A neural model of mechanisms of empathy deficits in narcissism	2013	Poland	Review	–	–		Neurophysiological aspects
Kang and Lakshmanan (57)	Narcissism and self-versus recipient-oriented imagery in charitable giving.	2018	Germany	Case- control study				Prosocial behavior
Kantrowitz (64)	Employing multiple theories and evoking new ideas: The use of clinical material.	2008	USA	Case report				Therapeutic implication
Kealy and Ogrodniczuk (5)	Narcissistic interpersonal problems in clinical practice	2011	USA	Review	–	–	–	Narcissism/empathy correlation
Kealy and Ogrodniczuk (11)	The narcissistic self and its psychological and neural correlates: An exploratory fMRI study.	2011	USA	Cross-sectional study	–	NI	fMRI data analysis; SCL-90-R; TAS	Neurophysiological aspects
Khodabakhsh and Besharat (31)	Mediation effect of narcissism on the relationship between empathy and the quality of interpersonal relationships.	2011	Iran	Cross-sectional study	EES	NPI	IIP	Prosocial behavior
Kleiger (69)	Emerging from the “dark night of the soul”: Healing the false self in a narcissistically vulnerable minister	1990	USA	Review	–	–	–	Therapeutic implication
Konrath et al. (34)	The relationship between narcissistic exploitativeness, dispositional empathy, and emotion recognition abilities.	2014	USA	Cross-sectional study	TEIQ; RMET; IRI	NPI	DAL	Narcissism/empathy correlation
Konrath et al. (60)	The strategic helper: Narcissism and prosocial motives and behaviors	2016	USA	Cross-sectional study	IRI	SINS; NPI	PTS; GSS; VFI	Prosocial behavior
Lehmann et al. (18)	The human and animal baby schema effect: Correlates of individual differences	2013	The Netherlands	Cross-sectional study	BES	NPI	ECR-r; IOS; NTB	Narcissism/empathy correlation
Luchner and Tantleff-Dunn (6)	Dysfunctional empathy in vulnerable narcissism	2016	USA	Cross-sectional study	IRI	NPI; HSNS	–	Narcissism/empathy correlation
Marcoux et al. (47)	Feeling but not caring: Empathic alteration in narcissistic men with high psychopathic traits	2014	Canada	Case-control study	IRI		PPI-R; QST; visual stimuli; tactile stimulation; electromyographic (EMG) and electroencephalographic (EEG) recordings	Narcissism/empathy correlation
Marissen et al. (8)	Disturbed emotion recognition in patients with narcissistic personality disorder	2012	The Netherlands	Case-control study	IRI	SCID-II	FRT	Narcissism/empathy correlation
Preston et al. (14)	Understanding empathy and its disorders through a focus on the neural mechanism	2020	USA	Review	–	–	–	Neurophysiological aspects
Ritter et al. (17)	Lack of empathy in patients with narcissistic personality disorder	2011	Germany	Case-control study	IRI; MET; MASC	SCID-II	GSI; SCL-90-R	Narcissism/empathy correlation
Roepke et al. (16)	Social cognition and emotional empathy in borderline and narcissistic personality disorder: Behavioral and fMRI data.	2010	USA	Case-control study	MET	–	MASC	Narcissism/empathy correlation
Ronningstam (30)	Beyond the diagnostic traits: A collaborative exploratory diagnostic process for dimensions and underpinnings of narcissistic personality disorder	2014	USA	Review	–	–	–	Narcissism/empathy correlation
Ronningstam (24)	Narcissistic personality disorder: A current review	2010	USA	Review	–	–	–	Narcissism/empathy correlation
Roepke (12)	Gray matter alterations in empathy-related brain regions of patients with narcissistic personality disorder	2012	Germany	Cross-sectional study	IRI	NPI	fMRI data analysis	Neurophysiological aspects
Szabó and Bereczkei (42)	Different paths to different strategies? Unique associations among facets of the dark triad, empathy, and trait emotional intelligence	2017	Hungary	Cross-sectional study	IRI; SREIT	NPI	MACH-IV; LSRP	Antisocial behavior
Thoma et al. (1)	Empathy and social problem solving in alcohol dependence, mood disorders and selected personality disorders.	2013	Germany	Review	–	–	–	Narcissism/empathy correlation
Topić Lukaèević and Bagarić (37)	Theoretical concepts of narcissistic personality disorder. Overview of narcissistic disorder in group analysis.	2018	Croatia	Review	–	–	–	Therapeutic implication
Urist (40)	Some structural considerations in the relationship between M and empathy.	1976	USA	Review	–	–	–	Narcissism/empathy correlation
van Mulukom et al. (68)	Broadening your mind to include others: The relationship between serotonergic psychedelic experiences and maladaptive narcissism	2020	United Kingdom	Retrospective Study	ECQ	NPI	AWE-S; EDI; IOSS; BSSS	Therapeutic implication
Vanaerschot (33)	It takes two to tango: On empathy with fragile processes.	2004	Belgium	Review	–	–	–	Narcissism/empathy correlation
Watson et al. (59)	Measures of the narcissistic personality: Complexity of relationships with self-esteem and empathy.	1992	USA	Cross-sectional study	IRI	NPI; OMNI	GIS; RSES	Narcissism/empathy correlation
Weise and Tuber (50)	The Self and object representations of narcissistically disturbed children: An empirical investigation.	2004	USA	Cross-sectional study	SCORS	Clinical interviews	–	Antisocial behavior
Yap et al. (53)	Cold hearts playing with fire: The dark triad, risk-taking, and empathy.	2021	Malaysia	Cross-sectional study	BES	–	SD3; DOSPERT	Narcissism/empathy correlation
Zimmerman (58)	The impact of perspective taking on the relationship between narcissism and affective empathy	2017	USA	Cross sectional study	IRI	PNI; NPI	–	Therapeutic implication

IAPS, the international affective picture system; SD3, short dark triad; TEQ, Toronto Empathy Questionnaire; PNI, pathological narcissism inventory; NPLC, narcissistic personality inventory for children; PCS, peer conflict scale; SSS, social support scale; MAI, the metacognition assessment interview; SCID-I, the structured clinical interview for DSM-IV axis I; SCID-II, the structured clinical interview for DSM-IV axis II; SCL90-R, the symptom checklist-90-r; TAS-20, the Toronto Alexithymia Scale; SOI, social orientation inventory; NPI, narcissistic personality inventory; SIS, Social Interest Scale; NPS, need for power scale; NTS, need threat scale; IRI, interpersonal reactivity index; NSS, Narcissism Spectrum Scale; ESCQ, emotional skills and competence questionnaire; TSIS, Tromsø Social Intelligence Scale; MET, Multifaceted Empathy Test; RPQ, reactive and proactive questionnaire; RSES, self-esteem scale; PES, psychological entitlement scale; NARQ, narcissistic admiration and rivalry questionnaire; ACME, affective and cognitive measure of empathy; RMET, “reading the mind in the eyes” test; ADP-IV, assessment of DSM-IV personality disorders questionnaire; DSCT, the digit symbol-coding test; QCAE, questionnaire of cognitive and affective empathy; IAS-A, indirect aggression scale–aggressor version; NI, narcissism inventory; EES, emotional empathy scale; IIP, inventory of interpersonal problems; TEIQ, trait emotional intelligence questionnaire; DAL, dictionary of affect in language; SINS, single item narcissism scale; DOSPERT, the domain-specific risk-taking; SCORS, Social Cognition and Object Relations Scale; GIS, Goal Instability and Superiority Scales; RSES, Rosenberg Self- Esteem Scale; ECQ, empathy components questionnaire; AWE-S, The Awe Experience Scale; EDI, the ego-dissolution inventory; IOSS, inclusion of other in the self-scale; BSSS, brief Sensation Seeking Scale; LSRP, Levenson Self-Report Psychopathy Scale; SREIT, self-report emotional intelligence test; MACH-IV, Measurement of Machiavellianism-IV; MASC, Movie for the assessment of social cognition; GSI, Global severity index; FRT, facial recognition task; PPI-R, psychopathic personality inventory; QST, a short quantitative sensory testing; BES, Basic Empathy Scale; ECR-r, Experiences in Close Relationships-revised; IOS, Inclusion of other in the self-scale; NTB, need to belong scale; HSNS, hypersensitive narcissism scale; PTS, Prosocial Tendencies Scale; GSS, General Social Survey; VFI, volunteer functions inventory; VIEQ, vulnerable isolation and enmity questionnaire.

The tests used to diagnose narcissistic personality disorder were the Structured Clinical Interview for DSM-IV Axis II (SCID-II), SCID-5-PD for the DSM-5, and the Pathological Narcissism Inventory (PNI) questionnaire. Empathy was evaluated with the Interpersonal Reactivity Index (IRI), the Multifaceted Empathy Test (MET), or the Toronto Empathy Questionnaire (TEQ).

Papers were sorted in different categories to facilitate in-depth analysis: narcissism and empathy correlation, antisocial behavior, neurophysiologic mechanisms, therapeutic implications and prosocial behaviors.

### Empathy

Empathy is both an emotional and cognitive construct influenced by the interplay between traits and environment.

Cognitive empathy is the capability to figure out someone else’s emotions and it is strictly related to the theory of mind (1). It implies the distinction between personal affective states and those of others. Reflections on personal thinking and on that of someone else is named “mindreading,” or “mentalizing,” and appears a semi-independent skill (2).

Affective empathy is correlated to acquaintance with emotions, elicited by emotional stimuli. Such a definition is incomplete, since it involves only positive aspects. Some authors explicitly argued that the observed empathic reaction should be congruent with that of the person they observe (3). On the contrary, empathic deficits in people with antisocial personality disorder entail dissonant or “contrast empathy” (4), when the subject experiences hate or even joy in a situation most people live with compassion or concern.

Kealy and Ogrodniczuk (5) proposed that the affective part is the key, while the cognitive factor is the pathway that creates such content.

Empathy also involves the ability of self-judgment and awareness of distinction between the self and other people, called “emotion regulation.” Such ability involves a governance on personal conduct and appropriateness to the social environment (6).

Several researches examined the most desirable correlates of emotion recognition capabilities, for example higher dispositional empathy (7, 8). Despite it, some authors admit that emotional competence can be directed antisocially, with manipulative connotations or drive others toward sociopathy and mischievous acts (9).

### Neurophysiological aspects of empathy

Some biological issues might be associated with those difficulties experienced by people affected by narcissistic personality disorder.

The primary brain structures involved in empathy are:

• the anterior insula (AI),
• the anterior cingulate cortex (ACC),
• specific regions of the medial prefrontal cortex (MPFC).

The AI and ACC are the principal intersections of the salience network (SN) (10), which chooses and organizes the flow of information from the internal and external receptors. This process might underpinning sentient awareness of feelings (11–13).

The AI might be a sort of switch center between two different networks of cognitive processing:

• the central executive network (CEN), linked to task execution.
• the default mode network (DMN), related to self-reflective processes (14).

The process, connected with affective empathy (“affective sharing”), implies the bottom-up evaluation of feelings that a subject feels in reaction to other people with equivalent feelings.

The “perception-action” model (15) explains it with a possible activation of similar brain zones [Anterior cingulate cortex (ACC) and anterior insula (AI)] in both observers and observed when the watcher examines or picture the feelings of someone they are evaluating.

On the other hand, the cognitive process of empathy is carried out by the prefrontal regions (16) and allows the observer to behave in a context-specific way.

Finally, the orbitofrontal cortex (OFC), the MPFC, the dorsolateral prefrontal cortex (DLPFC), and the ACC are involved in emotion regulation (6, 16).

Research highlights main obstacles both in the bottom-up pathway among narcissists, but the cognitive parts of empathy seem damaged as well (3, 17, 18).

Fan et al. (19) analyzed a group of non-clinical subjects, divided in high (HN) and low narcissism (LN). They were asked to empathize with images of faces expressing emotions. Evidence demonstrated a reduced deactivation of the right AI (rAI) and an increased activation of the posterior cingulate cortex (PCC), DLPFC, and premotor areas in reaction to non-emotional faces among HN people (17).

Furthermore, Jankowiak-Siuda and Zajkowski (3) examined the neurobiological roots of emphatic issues, linked them to a dysfunctional SN, determining an alteration in switching between the DMN and the CEN with an hyperactive faster DMN. The DMN is typically elicited during “mind wandering” or self-referential processing (20).

Since the insula is fundamental in the human threat detection system, NPs’ malfunction in the rAI could create disturbed estimation of some affective stimuli from the external world which are perceived as intimidating. Such an effect may increase sensitization with obstacles in moderating the response of the threat detection system (21).

Accordingly, NPs show a high degree of vulnerability to suffering, comparable to that of functional psychopaths (18).

### Empathy and narcissistic personality disorder

An interplay between narcissism and empathy was investigated from the clinical conceptualization of NPD to its launch in the DSM–III (22), deficits in empathy processing was considered a hallmark of pathological narcissism (23–26).

People affected by NPD describe themselves as superior but, at the same time, depend on and manipulate others to gain visibility and admiration (a reality called “narcissist supply”) (27).

In fact, a “look but do not touch” message is epitomic (28–30). Exploitation of other people does not imply meaningful contact with the subject affected by NPD. Therefore, the overt striving for social affirmation seems linked to a covert alienation.

Ritter et al. (17) demonstrated that people affected by NPD have issues in emotional, but not cognitive empathy, possibly because reading others’ emotions might be useful to reach personal purposes (31).

Subjects with high levels of narcissism declare lower degrees of perspective taking at the Interpersonal Reactivity Index, especially in questions about willingness to focus on empathic distress. Despite being able to perceive emotions like psychopaths (1, 32, 33), people affected by NPD may have compromised empathic functioning due to deficits in emotional empathy (e.g., neurobiological evidence) and motivation-based impairment in their cognitive empathic functioning.

Narcissism is a multifactorial construct, with several (e.g., entitlement, exploitativeness -E/E- and exhibitionism, self-sufficiency, superiority, vanity, leadership/authority) dysfunctional aspects (26).

Konrath et al. (34) explored the link between exploitation and skills of emotion recognition. They demonstrated that narcissists’ ability to read others’ emotions is driven by the trait E/E. Furthermore, exploitative people are more able at recognizing negative emotions because they look for vulnerability in others to find people to take advantage of and exploit (35).

A distinction between overt and covert narcissism is mandatory. Grandiose narcissism is characterized by entitlement, grandiosity and self-absorption with self-presentation under a favorable light by expressing superiority, aiming at dominance over others.

On the contrary, vulnerable narcissism is characterized by hypersensitivity, and dependence on others that reflects a fragile idea of self-worth which is regulated by strategies like diminishing the importance of connections to others (3).

Given-Wilson et al. (36), measured empathy, identity concerns, and interpersonal difficulties with the Interpersonal Reactivity Index–IRI. Covert narcissism seemed related to higher Personal Distress and Fantasy scores. High personal distress is linked with vulnerability and fearfulness (37). Vulnerable narcissism has been associated both with the fear of being taunted (gelotophobia) (leading to social retraction and isolation) (38), and with to the joy of making fun of others (katagelasticism) (emphasizing more antagonistic attitudes) (39, 40). On the other hand, Overt Narcissism was associated with lower personal distress, indicating affective detachment or unawareness of others’ feelings (2, 41).

The negative association between empathy and overt narcissism is based on disregarding others’ feelings, while the negative association with covert narcissism might be due to worries about themselves or more intense self-consciousness and may be overwhelmed by personal emotions, with failure in recognizing someone else’s perspectives (42).

### Dysfunctional aspects

Narcissistic personality disorder features indicate they do not have insufficient empathy, but that it is not efficient and subject to motivational and situational factors.

Narcissism is among malevolent traits of the Dark Triad (43), together with psychopathy and Machiavellianism. Further to this point, Gojković et al. (43) investigated correlations between Affective and Cognitive Measure of Empathy, admiration, rivalry, and the Short Dark Triad traits (SD3) (44). Rivalry, but not psychopathy, was the strongest trait of the dark core. Antagonism, embodied in rivalry, is the key part of callousness (45). Accordingly, rivalry predicts a lack of acceptable emotional response or recognition of someone else’s feelings, but also contradictory affects, a phenomenon called “affective dissonance.”

Intolerance toward emotions can play a role, since the subject might detect feelings in others, but that perception may arouse overwhelming power deprivation, shame or loss of internal control, thus stimulating aggressive responses or withdrawal (46). Such intolerance can coexist with reactivity to negative events and anticipation of humiliation (47) can coexist with emotional intolerance and issues in processing emotions, especially fear and shame, with reactive strategies of avoidance as well as defensive revengeful anger to regain control.

Furthermore, significant fluctuations in NPD empathic skills might be affected by self-regulation, increased when they feel confident and decreased when they are exposed or threatened (43).

### Antisocial behavior

It is crucial to understand the impact of narcissism on society and explore how to reduce antisocial behavior and improve prosocial ones.

Amiri and Behnezhad (48) highlighted that violent male offenders with “antisocial and narcissistic” traits have significant criminal careers. Vaughn et al. (49) showed that narcissistic items of the psychopathic personality inventory correlated with incarcerations and assaults in the previous 2 years (50). Johnson et al. (51) found that NPD symptoms in early adolescence prognosticate violent criminal behavior in mid-adolescence and early adulthood (52).

Narcissistic traits are escalating in western society with a 30% rise in the past 30 years (53) leading to increased criminal behavior with relevant public concern. People with a high level of narcissism respond aggressively toward a challenging source (54), presumably to regain self-esteem and dominance over others.

Beyond their motivation to aggressiveness or exploitation, it is questionable that a lack of empathy could be responsible for their impulsivity or devious plans, while disregard for others may support aggression as a response to perceived threats.

Barry et al. (54) demonstrated an inverse relation between empathy and aggression in narcissistic adolescents. Having some concern for others may result in a search for alternative strategies (e.g., manipulate others, self-aggrandizement) to reach social goals (55).

Grandiose narcissists may show overt empathic detachment, such as clear refusal, harsh criticism, and disapproval of others.

Therefore, when in a grandiose state, those empathic frailties may stimulate self-interests or competition.

Leaders with NPD can show both empathic issues and psychopathic, power motivated functioning, leading to illegal actions and active exploitations for personal gains (56).

Furthermore, Hepper et al. examined the effects of clinical and subclinical traits NPD on empathy in male prisoners compared to those with no criminal history. Being an offender is best predicted by entitlement, which is maladaptive in terms of antisocial behavior than NPD symptomatology.

Although lack of empathy gives a narcissist the “green light” to commit a criminal act, the initial feeling of deserving the best may also be crucial for narcissistic crimes (57).

### Prosocial behavior

Antisocial and prosocial behaviors are not antithetic. Prosociality might hide several reasons, even egoistic, such as receiving praise or attention, or having something in return (58). According to the Extended Agency Model (59) higher levels of narcissism are associated with more self-enhancement of qualities like intelligence and extraversion, but not agreeableness or morality (32, 60). This model affirms that narcissism intensifies the reward experienced from situations like having a social high status and power and, as a consequence, it leads to being more focused on success, power, and attention, and less on caring for others.

The prosociality of high narcissistic people is goal-directed to gain visibility and being ascribed as positive and talented. For example, they help people when others are watching but not anonymously. Moreover, they are likely to engage in “slacktivism” by posting online, despite donating money (61).

Accordingly, they can be labeled as strategic helpers, since they help others if they could help themselves in return (e.g., by receiving attention that implements narcissistic esteem).

### Therapeutic implication

Some theoretical models stress the core role of motivation as crucial in NPDs’ behavior and empathy, giving some room for change through psychotherapy.

Experts have different opinions about the best treatment approach, but patients affected by NPD are often considered resistant or even untreatable (62, 63).

A better analysis of the interplay we explored in this review aimed at stimulating awareness and more specific treatments.

Evidence suggested that the capacity for self-reflection and ability to think about someone else’s, sometimes called theory of mind or mind reading, are not the same thing but have reciprocal influence. Despite this, difficulties in one capacity predict difficulties in another (64).

Clinicians noted that patients with NPD have difficulty in facing their own emotions and in recognizing possible interpersonal reasons for their feelings (65). Moreover, self-awareness should be *a priority* to reach the awareness of others. Since narcissists see others as either alien or hostile, any attempt of mindreading before self-reflection probably is experienced as a request to “take the enemy’s part,” resulting in a stressful experience.

Instead, encourage self-reflectivity as first step may persuade patients to be more aware of their real attitudes, opposing to characteristics they simulate to achieve social acceptance (66).

Dialectical Behavior Therapy is based on the agreement that emotions might be frightful and at times, unbearable. This skills-based approach is recognized to support NPD people in determining their own needs and values and answer to responses from others appropriately (67). Furthermore, during therapeutic settings interpretations should be verbalized as questions or hypotheses, to facilitate the patient’s introspective interest and reduce negative responses.

Furthermore, since narcissists’ low empathy is induced by motivation, and, on that basis, simple perspective-taking instructions may be worth it in treatment.

When instructed to take the perspective of a suffering target person, the lack of empathy is lowered.

Consequently, addressing empathy in education, training, or public campaigns might be an efficient way to get to the heart of narcissists’ inadequacy (57).

Turning to talking about new perspective about drug therapy, based on a much less solid body of knowledge, van Mulukom et al. (68) showed that classical serotonergic psychedelic (CSP) drugs, thanks to induction awe and ego dissolution, may reduce of maladaptive NPD traits, such as a strong sense of entitlement and lack of empathy. The experience of ego-dissolution and lowered focus on the self, as induced by psychedelic drugs appear antagonistic to the self-focus and self-importance that is characteristic of high trait narcissism (68).

## Discussion

People affected by NPD show specific issues in empathy, but those difficulties are limited to its affective part. In fact, the cognitive portion seems preserved and essential for manipulative skill and exploitation of others.

Subjects with NPD may experience those problems with affective empathy because they feel others’ emotions as threatening and dangerous and react with detachment to preserve their own personal integrity. In addition to exploitation, a lack of empathic affectivity appears associated with proneness to criminal behaviors, particularly when NPD coexists with antisocial traits, contributing to psychopathy.

Furthermore, rivalry seems the key feature among the Dark Triad traits that supports callousness (44, 45) to its extreme pole embodied in “affective dissonance,” with contradictory affects in response to someone else’s feelings.

That alarming evidence, in terms of social implications and patient’s wellbeing, is often accompanied by poor therapeutic approaches. NPD patients are often labeled as untreatable, but self-reflection as a first and fundamental approach may represent a key step in facilitating the comprehension of someone else’s feeling and a crucial gateway to treatment.

### Limitations

Research on narcissistic personality disorder is limited. Patients affected by narcissistic personality disorder are often considered among the most difficult to be treated (62, 63). The fragility of their ego together with the tendency to impulsivity often obstruct the possibility of access to dynamic psychotherapy, which is considered the best treatment option. The crucial point in the treatment of NPD patients is their will to be treated (66), which is fundamental in psychotherapy. Such patients often consider treatments as a personal failure and refuse it.

Due to their label as untreatable, studies focused on the efficacy of psychotherapy in those patients are few and, consequently, those that analyze empathy and its correlates are even fewer.

Furthermore, most of the research is led on western populations, probably due to the rise of this illness in western cultures. This might represent an additional limitation because results cannot be generalized.

## Conclusion

Narcissistic traits are widespread in the contemporary Western population. Empathy plays a crucial role in both intrapersonal and interpersonal aspects of that personality disorder and influences both prosocial and antisocial behaviors.

Narcissism, although related to grandiose self and exploitativeness, is deeply associated with great personal suffering, vulnerability and correlates with important social consequences. Evidence of an ambivalent relationship between NPD and empathy, and the chance to work on therapy about this aspect, stress the importance of developing strategies to help patients with NPD to achieve a functional affective empathy.

Limits are many and consistent, but this manuscript aims at highlighting the evidence to date and stimulates further research due to the severity of this disorder and its spread in the general population, especially in the youngest part (adolescents and young adults).

## Author contributions

EdG planned the project, supervised the data, and literature analysis. OL and EA searched the database and analyzed the literature and data. EdG, OL, and EA wrote the manuscript. MC supervised the project. All authors approved the final version of the manuscript.
